# Engineered nanomaterials trigger abscopal effect in immunotherapy of metastatic cancers

**DOI:** 10.3389/fbioe.2022.890257

**Published:** 2022-10-26

**Authors:** Yuanliang Xia, Ruohan Yang, Jianshu Zhu, Hengyi Wang, Yuehong Li, Jiawei Fan, Changfeng Fu

**Affiliations:** ^1^ Department of Spine Surgery, The First Hospital of Jilin University, Changchun, China; ^2^ Cancer Center, The First Hospital of Jilin University, Changchun, China; ^3^ Department of Gastroenterology, The First Hospital of Jilin University, Changchun, China

**Keywords:** nanomaterials, immunotherapy, abscopal effect, metastatic cancer, immune cells

## Abstract

Despite advances in cancer treatment, metastatic cancer is still the main cause of death in cancer patients. At present, the treatment of metastatic cancer is limited to palliative care. The abscopal effect is a rare phenomenon in which shrinkage of metastatic tumors occurs simultaneously with the shrinkage of a tumor receiving localized treatment, such as local radiotherapy or immunotherapy. Immunotherapy shows promise for cancer treatment, but it also leads to consequences such as low responsiveness and immune-related adverse events. As a promising target-based approach, intravenous or intratumoral injection of nanomaterials provides new opportunities for improving cancer immunotherapy. Chemically modified nanomaterials may be able to trigger the abscopal effect by regulating immune cells. This review discusses the use of nanomaterials in killing metastatic tumor cells through the regulation of immune cells and the prospects of such nanomaterials for clinical use.

## 1 Introduction

Although many breakthroughs have been made in the clinical treatment of cancer, metastatic cancer remains extremely difficult to treat and is responsible for more than 90% of cancer deaths (Ganesh and Massagué, 2021). During metastasis, tumor cells penetrate blood vessels or lymph vessels, thus entering into circulation, and later extravasate from capillaries into organs distant from the original cancer site. Tumor cells then adapt to the new environment and develop into metastatic cancers (Mitchell et al., 2021). If a tumor cell metastasizes, the mobility of tumor cells becomes enhanced to the point that the cells easily penetrate the basement membrane and invade surrounding tissues. Sometimes, tumors metastasize despite treatment. At other times, cancer goes undiagnosed until after tumors have metastasized. The main treatment for metastatic cancer is currently palliative, and only 1–10% of metastatic cancers are surgically removed (Pagani et al., 2010). Since it usually cannot be cured, metastatic cancer is the main cause of death in cancer patients (Worrede et al., 2019).

Very rarely, local radiotherapy may cause the resolution of distant metastatic cancers that have not been treated with radiotherapy. This phenomenon is called the abscopal effect. The abscopal effect has been observed during the treatment of breast cancer (Deng et al., 2020a), melanoma (Yang et al., 2020), lung cancer (Perry et al., 2020), pancreatic cancer (Sun et al., 2020a), and other malignant tumors. Although the mechanism of the abscopal effect is still unknown, it has been proven that the abscopal effect is related to the immune mechanism (Ngwa et al., 2018).

Cancer immunotherapy is considered to be a promising strategy for curing metastatic cancer by restoring or strengthening a patient’s immune system (Cable et al., 2021). Unlike radiotherapy and chemotherapy, immunotherapy does not target tumor cells but rather the body’s immune system. Therefore, it is important to understand the relationship between tumor cells and the immune system. The process of adaptive and innate immune systems controlling tumor growth and shaping tumor immunogenicity is called tumor immunoediting (Desai et al., 2022). Immunoediting occurs in three stages: elimination, equilibrium, and escape (Desai et al., 2022). In this process, the immune system acts as a tumor stimulant and tumor suppressor (Saadeldin et al., 2021). During the growth process, tumor cells avoid being killed by the immune system through immune checkpoints, thereby proliferating in large numbers to form cancer (Tsuchiya and Shiota, 2021). After an immunotherapy drug enters the body, it restores the ability of immune cells to recognize cancer cells so that the immune cells kill cancer cells in large quantities to achieve the purpose of treatment. In the last 2 decades, cancer immunotherapy development has included the creation of vaccines, monoclonal antibodies, and small immunomodulatory molecules (Gu et al., 2020). Cancer immunotherapy improves a patient’s immune function and avoid the decline in immune function caused by surgical stress, thereby greatly improving the success rate of surgical treatment.

There are two main purposes of cancer immunotherapy. One is to directly modify immune cells to stimulate the activity of immune cells against tumors, and the other is to reduce the immune suppression of tumor cells or the microenvironment to improve immune cell activity through small molecules or antibody regulation (Cable et al., 2021). However, not all cancer cells are recognized and eliminated by the immune system (Li et al., 2020a). Immune checkpoints expressed on immune cells are a class of molecules that regulates immune activation. Their presence prevents attacking on their own tissues due to the strengthening of the immune system. However, the corresponding receptors are also expressed on tumor cells, which prevents the immune system from killing tumor cells (Zhou et al., 2020a). Although immune checkpoint inhibitors disrupt the balance between immune cells and tumor cells, they also have a serious consequence: immune-related adverse events (irAEs) (Zhou et al., 2020a). IrAEs arise from inflammatory overreactions, usually involving endocrine glands, caused by immune enhancement. These inflammatory overreactions typically induce dermatitis, colitis, and thyroiditis, but they also cause rarer conditions with high mortality, such as myocarditis, myositis, and encephalitis (Hommes et al., 2021). Hence, irAEs are a major obstacle to the widespread use of cancer immunotherapy (Li et al., 2020a; Gomes and Franco, 2022).

**SCHEME 1:**
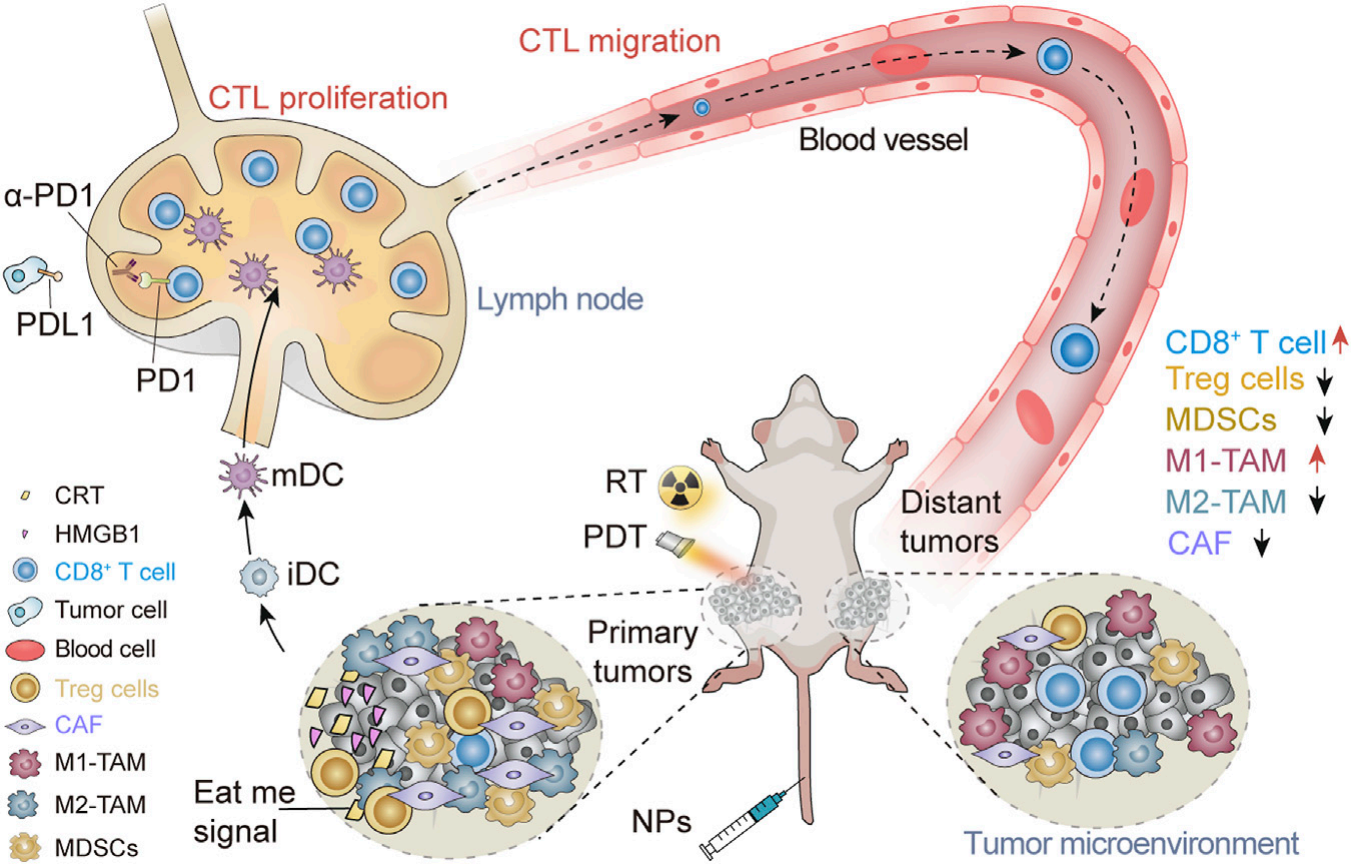
Schematic illustration of nanomaterials triggering the abscopal effect in the immunotherapeutic treatment of metastatic cancer. Nanomaterials trigger the abscopal effect in immunotherapy combined with RT and PDT, causing the increase of CD8^+^ T cells and M1-TAMs in the distant TME and the decrease of Tregs, MDSCs, M2-TAMs, and CAF.

Nanomaterials provide new opportunities for improving the efficacy of cancer immunotherapy and reducing its adverse reactions. Because of characteristics such as their size, electrical properties, and shape, nanoparticles are sensitive to chemical modification (Nasirmoghadas et al., 2021). Nanomaterials can penetrate abnormal extracellular matrices, vascular endothelial cells, and complex tumor environments to target tumor cells precisely (Stephen and Zhang, 2021; Thangam et al., 2021). Subsequently, nanoparticles accumulated at a tumor site because of the enhanced permeability retention (EPR) of tumor cells. EPR enhanced the release of nanoparticles around tumor cells, thus greatly improving treatment effectiveness. However, the EPR effect alone cannot accumulate enough nanoparticles because of the high fluid pressure in the tumor stroma (Fu and Xiang, 2020). Nanoparticles can be modified *via* targeting and biological reactivity (Mitchell et al., 2021), such as the pH response and hypoxia response. Active targeting through surface modification of nanoparticles improved the recognition of tumor sites, thereby increasing the drug concentration in the tumor microenvironment (TME). Chemically modified nanomaterials have good biological tissue compatibility which reduces the concentration of cytokines or deliverables directly leaked into the bloodstream, thereby reducing the occurrence of irAEs (Huang et al., 2021). Although the details of the mechanism of the abscopal effect are unknown, we have found that immune cells play an integral role in the abscopal effect. This review discusses the roles of different types of immune cells and nanomaterials in the abscopal effect (Scheme 1 and Table 1). We look forward to the prospects for the application of the abscopal effect in the clinical treatment of metastatic tumors.

**TABLE 1:** Nanomaterials trigger abscopal effect for immunotherapy of metastatic cancers.

Function	Type of material	Therapeutic components	Conventional treatment	Cancer	Result	References
DCs recruit	The core-shell gold nanocage@manganese dioxide	Manganese dioxide	Photodynamic therapy	Breast cancer	The core-shell gold nanocage@manganese dioxide offers a promising approach to ablate primary tumor and simultaneously prevent tumor metastases *via* immunogenic abscopal effects	Liang et al. (2018a)
	Hybrid protein oxygen nanocarrier	Photosensitizer	Photodynamic therapy	Breast cancer	Hybrid protein oxygen nanocarrier -mediated immunogenic PDT could destroy primary tumors and effectively suppress distant tumors and lung metastasis by evoking systemic anti-tumor immunity	Chen et al. (2018)
	pH-responsive nanovesicles	Photosensitizer	Photodynamic therapy	Melanoma	The nanocarrier to induce ICD for the host’s immunity activation	Yang et al. (2020)
	Maleimide liposome	Maleimide	Photothermal therapy	Breast cancer	The therapeutic systems improved the infiltration of CD8^+^ T cells to 53% in tumor tissues, eliciting strong abscopal effect and antimetastasis effect	Zhou et al. (2020b)
	PEG-PLA hydrogels	CCL21	—	—	PEG-PLA hydrogels are able to recruit specific immune cell populations to injection sites	Fenton et al. (2019)
Mature DCs	Polysaccharide nanoparticles	TLR agonists	Radiotherapy	Breast cancer	Polysaccharide nanoparticles can reverse TEM and enhance the radiation induced abscopal effect	Pang et al. (2019)
	Cationic nanoscale metal–organic framework	CpG	Photodynamic therapy	Breast cancer	Cationic nanoscale metal–organic framework robust abscopal effect with >97% tumor regression in a bilateral breast cancer model	Ni et al. (2020)
	PEG-based NPs	CpG	Intratumoral injection	Lung Cancer	PEG-based NPs are less toxic and reduce tumor burden in a mouse model of metastatic lung cancer	Perry et al. (2020)
	Cationic nanoscale metal–organic framework	CpG	Photodynamic therapy	Breast cancer	It provides anticancer efficacy and abscopal effect with >97% tumor regression in bilateral breast cancer models	Ni et al. (2020)
	Prussian blue nanoparticles	CpG	Photothermal therapy, Radiotherapy	Neuroblastoma	Nanoparticles carrying CpG activate DCs and trigger abscopal effects to resolve metastases and enable mice to obtain long-term immunity	Cano-Mejia et al. (2020)
	Self-adjuvanted molecular activator (SeaMac) nanovaccines	Maleimide	Photodynamic therapy	Melanoma Colon cancer	Nanovaccines inhibit tumor growth significantly and prolong the survival of tumor-bearing mice	Luo et al. (2021)
	Light-activable immunological adjuvant (LIA)	Chlorin e6	Photothermal therapy	Breast cancer	The LIA efficiently inhibits both primary and abscopal tumour growth and induces strong antigen-specific immune memory effect to prevent tumour metastasis	Wang et al. (2021a)
				Colorectal Cancer		
	Cisplatin loaded nanoparticles	Pt	Radiotherapy	Breast cancer	Cisplatin loaded nanoparticles can amplify RT-induced immune activation and break through the efficiency limitation of the RT plus anti-PD1 induced abscopal effect	Wang et al. (2021b)
	Nanofluidic drug-eluting seed	CD40	Radiotherapy	Breast cancer	Nanofluidic drug-eluting seed boosted the abscopal effect towards attenuating lung metastatic burden	Liu et al. (2020)
		TLR agonists				
Block immune checkpoints	Celastrol nanoemulsion	Celastrol (CEL)	—	Melanoma	Celastrol nanoemulsion retaining a high tumor CEL concentration activated the immune system efficiently, which inhibited both the treated tumor and the distant untreated tumor	Qiu et al. (2021)
	Upconversion nanoparticles	Chlorin e6 (photosensitizer)	Photodynamic therapy	Colorectal Cancer	Upconversion nanoparticles promote strong anti-tumor immune responses, inhibit the growth of distant tumors and prevent tumor reoccurrence *via* the immune memory effect	Xu et al. (2017)
	ROS-sensitive lipid-polymer hybrid nanoparticles	Doxorubicin	Photodynamic therapy	Breast cancer	The cascade chemo-PDT could evoke anticancer immune responses to generate an abscopal effect, which could simultaneously inhibit primary and distant tumor growth	Hu et al. (2019)
	Prussian blue (PB) nanoparticle	Sorafenib	Photothermal therapy	Hepatocellular carcinoma	The NPs displayed promising inhibitory effects on tumor metastasis and recurrence and produced an abscopal effect and long-term immunological memory	Zhou et al. (2020c)
	Nanoliposome loaded with PhA	pheophorbide A	Photodynamic therapy	Melanoma	Nanoliposome loaded with PhA induce strong abscopal tumor suppression and induction of robust systemic immune responses	Hwang et al. (2020)
	Tandem peptide nanocomplex	CpG DNA ligand of TLR9	—	Melanoma	The Tandem peptide nanocomplex can drive accumulation of cargoes in tumors in a manner dependent upon their homing properties	Buss and Bhatia, (2020)
	Copper chalcogenide nanoparticles	TLR9 agonists	Photothermal therapy	Melanoma	Copper chalcogenide nanoparticles synergizes Toll-like receptor 9 agonists and immune checkpoint inhibitors to enhance the abscopal effect in tumors	Cao et al. (2020)
Block the IDO	Mesoporous silica nanoparticles	Doxorubicin, IDO inhibitor	Chemotherapy	Glioblastoma	DCs were vastly recruited and the cytotoxic T cells were significantly activated	Kuang et al. (2018)
	Chitosan nanoparticles	IDO inhibitor	Photothermal therapy	Lung cancer	This synergistic strategy significantly inhibited lung metastasis and controlled the development of already metastasized tumors	Chen et al. (2020)
	Polydopamine—coated nanoparticles	IDO inhibitor	Photothermal therapy	Pancreatic cancer	Polydopamine -coated nanoparticles result in a significant growth inhibition of both primary tumor and the unirradiated distal tumor	Sun et al. (2020a)
	Mesoporous silica nanoparticles	Doxorubicin, IDO inhibitor	Chemotherapy	Breast cancer	Mesoporous silica nanoparticles potentiate both tumor local and systemic anti-tumor immunity, suppressing primary tumor growth by 78% with an 83% reduction in metastatic foci	Li et al. (2020b)
				Colon cancer		
	Redox-activated liposome	IDO inhibitor	Photodynamic therapy	Breast cancer	The nanovesicle achieve effective inhibition of tumor growth and showing the efficacy in inhibiting primary and distant tumors	Liu et al. (2019)
Deliver cytokines	Photothermal agent and gene co-delivery nanoparticle	Plasmid encoding IL-12 gene	Photothermal therapy	Melanoma	Nanoparticles could significantly induce systemic immune responses to efficiently eliminate possible metastatic lesions through abscopal effects	Lin et al. (2021)
	Polymetformin nanoparticles	Plasmid encoding IL-12 gene and Doxorubicin	—	Breast cancer	Polymetformin nanoparticles exhibit excellent anti-tumor activity and lung metastasis inhibition *via* DOX/pIL-12-mediated chemoimmune synergy	Sun et al. (2020b)
	Layer-by-layer nanoparticles	IL-12	—	Ovarian cancer	Layer-by-layer nanoparticles reduce systemic toxicity without compromising the efficacy of IL-12 therapy	Barberio et al. (2020)
	Chitosan based nanoparticles	Doxorubicin,IL-12	—	Hepatoma cancer	The combinational administration of DOX and rhIL-2 based on polymer nanoparticles could serve as an effective strategy in antitumor therapy	Wu et al. (2017)
	Nanoscale liposomal polymeric gels	IL-2 and TGF-β receptor-I inhibitors	—	Melanoma	Nanoscale liposomal polymeric gels releasing TGF-β inhibitor and IL-2 significantly delayed tumour growth, increased survival of tumour-bearing mice	Park et al. (2012)
	PEG nanoparticles	IL-2 receptor agonist	—	Breast cancer Pancreatic cancer	PEG nanoparticles mediate selective Treg depletion of intratumoral but not peripheral Tregs	Sharma et al. (2020)
Deplete Tregs	Curcumin analog nanoparticle	Treg cell specific antibody (mAb)	Chemotherapy	Breast cancer	Curcumin analog nanoparticle reduce the production of Treg cells by inhibiting the expression of foxp3 and amplifie the role of chemotherapy in metastatic breast cancer	Du et al. (2020)
	Polydopamine- Indocyanine green nanoparticles	Catalase	Photothermal therapy	Breast cancer	Polydopamine- Indocyanine green nanoparticles inhibition ratio of 95.1% for primary cancers and 68.7% for abscopal cancers in breast cancer-bearing mice	Sun et al. (2021)
		anti-GITR antibody (DTA-1)				
	CaO2 nanoparticle	CaO2	Sonodynamic therapy (SDT)	Pancreatic cancer	Nanoparticle increase in tumour cytotoxic T cells (CD8^+^) and a decrease in immunosuppressive tumour regulatory T cells	Nicholas et al. (2021)
Block MDSCs infiltration	Phenylboronic acid modified nanoparticles	P-selectin glycoprotein ligand-1	Photothermal therapy	Pancreatic cancer	Phenylboronic acid modified nanoparticles could significantly improve the immune microenvironment of pancreatic tumor and inhibit spontaneous metastases	Lu et al. (2021)
	Nanoscale metal-organic frameworks	phenylboronic acid	Radiotherapy -radiodynamic therapy	Breast cancer	Nanoparticle led to robust abscopal effects and significant antimetastatic effects	Ni et al. (2019)
Induce polarization of macrophages polarize	β-cyclodextrin nanoparticles	TLR9 agonists	—	Glioblastoma	β-cyclodextrin nanoparticles alters the functional localization of the tumor immune microenvironment towards the M1 phenotype, thereby controlling tumor growth	Rodell et al. (2018)
	Cell-mediated delivery system	Apoptotic body	Photothermal therapy	Breast cancer	The cell-mediated delivery system can not only efficiently ablate primary tumors but also elicit a potent immunity to prevent tumors from metastasizing and recurring	Zheng et al. (2020a)
	Hyaluronic acid nanoparticles	MnO2	Chemotherapy	Breast cancer	Targeted delivery of Hyaluronic acid nanoparticles to TAM can successfully alleviate tumor hypoxia and enhance chemotherapy response by polarizing TAM from M2 type to M1 type	Song et al. (2016)
	Polyethylene glycol-polyglutamic acid nanoparticles	Macrophage colony-stimulating factors	—	Lung cancer	Nanoparticles can successfully repolarize TAM to the M1 phenotype with important implications in anticancer immunotherapy	Mao et al. (2019)
Repolarizing macrophages into tumors	Cationic Polymeric Nanoparticle	CCR2 siRNA	—	Breast cancer	Cationic nanoparticles can more effectively alter the immunosuppressive tumor microenvironment and exhibit excellent antitumor effects	Shen et al. (2018)
	Bi-based mesoporous upconversion nanophosphor	Doxorubicin	Radiotherapy	Lung cancer	This study opens the door to further enhance the abscopal effects and inhibit the metastasis in radiotherapy	Qin et al. (2020)
Regulation of CAF	The oxidized nanoparticles	—	Hyperthermia	—	Nanoparticles exhibit rapid and efficient cellular internalization against fibroblasts with low cytotoxicity and high induction of cell death	Ferraz et al. (2020)
	Navitoclax -loaded functionalized nanocages	Navitoclax		Colon cancer Melanoma	Navitoclax -FAP provided selective targeting of FAP-overexpressing fibroblasts over cancer cells and proved more effective in killing target fibroblasts	Sitia et al. (2021)
	PLGA nanoparticles	Perfluorocarbons	Radiotherapy	Breast cancer	PLGA nanoparticles significantly enhances the radiotherapeutic effect on local tumors and also inhibits the growth of remote tumors by an enhanced abscopal effect	Duan et al. (2021)
Regulate NK cells	NK Cell-Membranes-Cloaked Nanoparticles	Benzoic acid	Photodynamic therapy	Breast cancer	NK Cell-Membranes-Cloaked Nanoparticles selectively were able to eliminate primary tumor growth and produce an abscopal effect to inhibit distant tumors	Deng et al. (2018)

## 2 Nanomaterials for regulating dendritic cells to trigger abscopal effect

Dendritic cells (DCs) are important antigen-presenting cells (APCs) in the body. DCs cross-present the antigen of major histocompatibility complex (MHC) class I molecules to activate CD8^+^ T cells. Tumor-associated antigens (TAAs) can be recognized, processed, and presented by DCs. However, immature DCs produce regulatory T cells (Tregs) to inhibit the activity of T cells (Wculek et al., 2019). Therefore, the number of mature DCs in the body is too low to cause a strong immune response. CD80 and CD86 are markers of mature DC surfaces. Nanomaterials are used to transport tumor antigens, immune adjuvants, and exogenous DCs to tumor-draining lymph nodes (TDLNs), enhance the activity of DCs, and cause a powerful antigen presentation response (Morales-Orue et al., 2019).

### 2.1 Nanomaterials for recruiting dendritic cells

Antigen presentation in the body is often due to an insufficient number of DCs, which results in a failure to cause more CD8^+^ T cells to infiltrate the tumor (Gardner et al., 2020). Low immunogenicity and weak immune response limit abscopal effects in the treatment of metastatic tumors. Immunogenic cell death (ICD) promote the recruitment of DCs in metastases. Min et al. (2017) developed several antigen-capturing nanoparticles (ACNPs) based on poly (lactic-co-glycolic acid) (PLGA). Research suggests that positively charged particles were more likely to be captured by DCs. ACNPs delivered TAAs to APCs and promoted DCs trafficking to TDLNs to enhanced antitumor immune responses. In addition to TAAs, high-mobility group box 1 (HMGB1), damage-associated molecular patterns (DAMPs), and histone proteins have also been shown to promote the recruitment of DCs (Ren et al., 2018; McCaw et al., 2019; Wang and Zhang, 2020). Natural compounds often act as carriers to transport these substances around tumors. Because of its large surface area, large pore size, multifunctional surface modifiability, and biodegradability, silica has been widely used for drug delivery (Nguyen et al., 2020a). Deng et al. (2020b) developed silica mesoporous nanotechnology for the delivery of nanovaccines for metastatic cancer immunotherapy. The silica nanoparticles enhance the recruitment of DCs. However, whether the nanoparticles trigger the abscopal effect has not been verified.

Tumor cells are surrounded by a hypoxic environment, which is conducive to tumor cells escaping attack by the immune system (Fu and Xiang, 2020). Therefore, increasing the amount of oxygen in the tumor environment is an effective way to induce ICD and activate DCs to produce powerful anti-tumor effects. Gold nanocages (AuNCs) have attracted considerable attention because of their hollow porous structures and controllable optical performance. Liang et al. (2018a) encapsulated manganese dioxide with AuNCs (AuNC@MnO _2_) for targeting tumor-associated macrophages (TAMs) for oxygen production. AuNC@MnO _2_ was prepared by mixing AuNC and polyethylene glycol (PEG) in a potassium permanganate solution by magnetic stirring. With an average size of 91 nm and a surface charge of + 5.6 mV, AuNC@MnO_2_ had the advantage of reactive oxygen species (ROS) responsiveness to generate oxygen around the tumor. The nanocages elicited ICD by provoking dying tumor cells to induce enhanced exposure and release of DAMPs, such as calreticulin, adenosine triphosphate (ATP), and HMGB1. DAMPs then promoted the recruitment of DCs and increased the antigen engulfment and presentation, followed by prominent activation of effector cells (e.g., CD8 T cells, CD4 T cells, and NK cells) for inhibition of primary tumor and lung metastases. AuNC@MnO _2_ combined with photodynamics up-regulated the expression of CD80 and CD86 up to 7.5% and reduced the number of lung metastases in tumor-bearing mice (Liang et al., 2018a). Yang et al. (2020) explored PEG cationic peptides to self-assemble into pH-responsive nanovesicles. PEG nanovesicles with a hydrodynamic diameter of 55 nm. *In vitro* experiments showed that PEG nanovesicles were endocytosed by tumor cells within 4 h. Encapsulation of photosensitizers into PEG nanovesicles generated ROS within tumors to induce tumor cell death. In tumor-bearing mice, PEG nanovesicles significantly inhibited the growth of primary and distant tumors within 17 days. Human serum albumin (HAS) shows natural biocompatibility, excellent stability, and positive tumor targeting ability. Chen et al. (2018) utilized reduced HAS and hemoglobin (Hb) through intermolecular disulfide hybridization to obtain hybrid protein oxygen carrier (HPOC) nanoparticles. The advantage of HPOC was that Hb’s own oxygen-carrying ability provided oxygen to the hypoxic environment of tumor cells and improved the treatment efficiency. In another study, Zhou et al. utilized maleimide liposomes to deplete intracellular glutathione. Liposomes remodeled TAMs to promote DCs recruitment (Zhou et al., 2020b).

CCL21 is a chemokine that promotes the recruitment of DCs (Hu et al., 2020). Owen et al. developed a hydrogel made of PEG-polylactic acid (PLA) for carrying CCL21 to promote DCs recruitment (Fenton et al., 2019). The advantage of hydrogel was that after injection into the mouse, CCL21 was released within 48 h to recruit DCs to the injection site. In another study, protamine sulfate loaded with seaweed shown high loading rate of CCL (Poelaert et al., 2020). Protamine sulfate acts as a polycation, stabilizing alginate nanoparticles into polyelectron complexes. Intratumoral injection of nanoparticles caused neuroblastoma to decline and improved the long-term immune effect of mice (Poelaert et al., 2020). However, no instances of the abscopal effect being triggered by CCL21 loaded on nanomaterials have yet been reported. We believe that nanomaterials to induce CCL21 to enter the metastatic tumor site to recruit DC is a promising method for metastatic tumor treatment.

### 2.2 Nanomaterials for eliciting DC maturation

Immature DCs cause T cells to be unresponsive to tumors and lead to immune tolerance (Dudek et al., 2013). Therefore, the maturation of DCs is crucial to achieving the abscopal effect in immunotherapy. Because of the presence of transforming growth factor-β (TGFβ) and interleukin 10 (IL10), the number of DCs in tumor patients is abnormal (Brown et al., 2001). Toll-like receptors (TLRs) are an important class of protein molecules involved in non-specific immunity. TLR9 is mainly expressed in B cells and plasmacytoid dendritic cells (pDCs) (Krieg, 2007). The activation of TLR9 on pDCs has a variety of effects, such as the expression of Interferon-γ (IFN-γ), Th1-type cytokines (tumor necrosis factors-α, IL-2), TNF-related apoptosis-inducing ligand (TRAIL), and co-stimulatory molecules (CD80, CD86) (Suek et al., 2019). TLR7 and TLR9 have been proven to be related to the maturity of DCs (Leong et al., 2019).

Natural polysaccharides are polymers with good biological safety and various biological functions (Zhu et al., 2016; Yu et al., 2018). Natural polysaccharide nanoparticles carrying TLR agonists caused more DCs activation (Pang et al., 2019). Although the application of TLR agonists has a good effect, it once caused obvious inflammation. Darling et al. (2020) explored acetic anhydride nanoparticles with a mixture of 3,6-dioxaoctante (CPTEG) and 1,6-bis (p-carboxyphenoxy) hexane (CPH) by a molar ratio of 20:80. The average particle size of the nanoparticles was approximately 200 nm. Acetic anhydride nanoparticles provided antigen delivery and DCs activation while avoiding the extensive inflammatory response usually associated with traditional adjuvants (Darling et al., 2020). Nanomaterials carrying TLR agonists have been reported to enhance DCs activation and trigger the abscopal effect (Cano-Mejia et al., 2020; Ni et al., 2020; Perry et al., 2020; Luo et al., 2021). For example, as a class of molecular nanomaterials, cationic nanoscale metal–organic frameworks (nMOFs) are suited for biomedical applications because of their crystallinity, tuneability, and porosity (Ni et al., 2020). nMOFs had emerged as novel nanophotosensitizers for photodynamic therapy (PDT) with high photosensitizer (PS) loadings, enhancing the abscopal effect of PDT triggering. Prussian blue (PB) also shows good biocompatibility and is widely used in biomedicine. Rohan et al. developed PB nanoparticles for the delivery of CpG and promoted the maturation of DCs in neuroblastoma (Cano-Mejia et al., 2020). PB nanoparticles alter the presence of immunomodulatory receptors and ligands on the treated tumor cells and generates an abscopal effect. PB exhibits pH-dependent biodegradability, mitigating concerns over long-term persistence and toxicity within the body (Cano-Mejia et al., 2020). The study found that the local release of ROS from the tumor also promoted the maturation of DCs. Wang et al. (2021a) devoloped light-activable immunological adjuvant (LIA) loaded with chlorin e6, after being internalized by tumor cells in a mouse model of breast cancer. LIA produced ROS under light to induce tumor cells to released antigens and also promoted DCs maturation.

**FIGURE 1:**
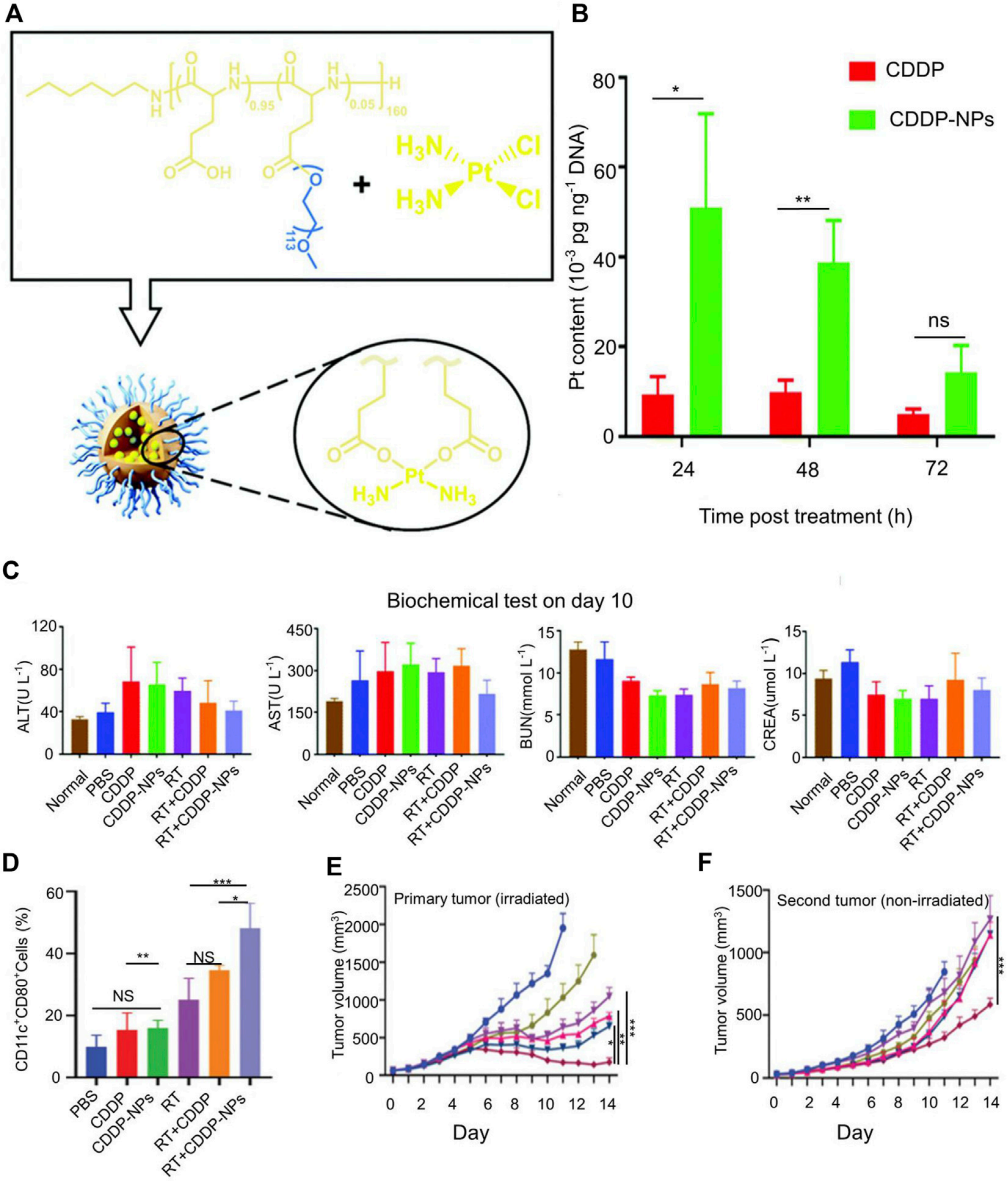
Cisplatin nanoparticles boost abscopal effect. **(A)** Structure of cisplatin (CDDP) loaded poly (l-glutamic acid)-graft-methoxy poly (ethylene glycol) complex nanoparticles (CDDP-NPs). **(B)** The Pt content in the tumor DNA of differentially treated mice on the basis of CDDP for 24, 48, and 72 h. **(C)** Serum levels of alanine aminotransferase (ALT), aspartate aminotransferase (AST), blood urea nitrogen (BUN) and creatinine (CREA) in the indicated groups on day 10. **(D)** The population of activated DCs in tumors was evaluated on day 9 *in vivo*. **(E)** The tumor volume of primary tumor. **(F)** The tumor volume of distant tumor. Reproduced with permission from (Wang et al., 2021b).

Radiotherapy (RT) can break the DNA double bond of tumor cells, release TAAs, and cause tumor cells to produce ICD. RT has been widely used clinically. However, RT is normally used for localized treatment rather than to treat metastatic cancer, and it is not safe for certain areas of the body. Chen et al. explored cisplatin loaded within PLGA-graft-methoxy PEG complex nanoparticles (CDDP-NPs) (Figure 1) (Wang et al., 2021b). Platinum crosslinks the DNA double strand, reducing the stability of DNA by a process called DNA platination. This nanomaterial enhanced the body’s tolerance to radiation doses and caused more DNA damage in tumor cells. CDDP-NPs resulted in greater DNA platinization compared to CDDP at 24 h (51.02 × 10^–3^ pg Pt per ng DNA), 48 h (38.80 × 10^–3^ pg Pt per ng DNA), and 72 h (14.24 × 10^–3^ pg Pt per ng DNA) (Figure 1B). Figure 1B The nanoparticles extended the residence time of platinum-based chemotherapeutics around the tumor but did not damage the cells of the whole body (Figure 1C). The combined application of cisplatin nanoparticles and radiotherapy increased the expression of CD80 in distant tumors (Figure 1D), boosted the abscopal effect and delayed the progression of primary and distant tumors (Figures 1E,F). However, CDDP-NPs induced hematopoietic suppression and peripheral cytopenias (Wang et al., 2021b). This may be detrimental to the application of CDDP. Therefore, further research is needed to reduce its side effects.

CD40/CD40L is another pathway that causes the maturation of DCs. CD40 increase the expression of immunoglobulin superfamily co-stimulatory molecules such as CD80 and CD86 (Vonderheide, 2020). CD40 signaling occurs through two adaptor proteins, TNFR-related factor (TRAF) and Jak family kinase 3 (JAK3). These proteins activate various signaling pathways, including MAPK, PI3K, and NF-kβ, which in turn cause DCs to mature. It has been confirmed that CD40 agonists boost abscopal effects by activating DCs (Wood et al., 2020). Although CD40 affects the maturation of DCs, CD40 stimulation alone does not enable mice to obtain long-term immunity but rather produces ineffective cytokines and specific T cells (Lau et al., 2020). The combination of CD40 antibodies and TLR agonists is generally more effective. Liu et al. developed nanofluidic drug-eluting seeds (NDES) for the delivery of CD40 antibodies and TLR agonists into tumors (Liu et al., 2020). The nanochannel of NDESs was 150 nm, and intratumoral injection of NDESs continuously and slowly released CD40 as long as 14 days. In combination with external radiation therapy, NDESs enhanced the abscopal effect and reduced the chance of metastasis by promoting the maturation of DCs.

In conclusion, some nanomaterials targeting DCs exert anti-tumor effects. Nanomaterials located with TLR agonists not only treat existing tumors but also play a role in preventing tumor metastasis. Not only that, nanomaterials have been proved to good biological tissue compatibility and targeting. Many effects, such as regulate DC maturation and activation, have been shown to exist simultaneously. These findings have opened up new avenues for progress in tumor treatment.

## 3 Nanomaterials for regulating T cells to trigger abscopal effects

Once the MHC on a DCs binds to a T cells receptor, the T cells will be activated and differentiate into effector T cells. Effector T cells are mainly composed of CD4^+^ effector T cells and CD8^+^ T cells. CD8^+^ T cells are the main anti-tumor cells in the body (Gardner et al., 2020). Subsequently, CD8^+^ effector T cells circulate systemically, infiltrate tumor lesions through interstitial blood vessels, and kill metastatic tumor cells (Tsuchiya and Shiota, 2021). However, many inhibitory factors in the TME hinder T cells’ function.

### 3.1 Nanomaterials with blocking immune checkpoints

Immune checkpoints are inhibitory regulatory molecules in the immune system that are essential for maintaining self-tolerance and preventing autoimmune reactions. Immune checkpoints expressed on immune cells inhibit the function of immune cells so that the body does not produce enough effective anti-tumor immune responses. The immune checkpoint blockade (ICB) enhances anti-tumor activity by disrupting inhibitory T cells signaling (Shi et al., 2020). Programmed cell death protein 1 (PD-1) is an important immunosuppressive transmembrane protein expressed on the surface of T cells. PD-L1 is a ligand of PD-1. The combination of PD-1 on the surface of T cells with PD-L1 will transmit inhibitory signals and reduce the proliferation of CD8^+^ T cells in lymph nodes. One of the important mechanisms by which tumor cells evade the attack of immune cells is by expressing PD-L1 (Han et al., 2020). Metastatic cancer cells express more PD-L1 than normal tissue cells (Eckert et al., 2018). Blocking the PD-1/PD-L1 signaling pathway can thus enhance the killing of metastatic tumors by CD8^+^ T cells. In addition, cytotoxic T lymphocyte-associated antigen-4 (CTLA-4) is a leukocyte differentiation antigen, a transmembrane receptor on T cells, and it shares the B7 molecular ligand with CD28. CD28 is a transmembrane protein on the surface of T cells. The APC surface expresses B7.1 (CD80) and B7.2 (CD86) ligands. The combination of CD28 and B7 molecular ligands can activate T cells. CTLA4 has a structure homologous to CD20 that can compete with CD28 for B7 ligands, resulting in insufficient T cell activation (Hosseini et al., 2020). Although ICB has achieved good results, it only works on tumor cells that have been pre-infiltrated by T cells (Spranger and Gajewski, 2018). In metastatic tumors, insufficient T cell infiltration has become a major obstacle to treatment. Therefore, how to induce the infiltration of T cells into metastatic TME and increase T cells’ activity have emerged as key problems in the pursuit of curing metastatic cancer.

Blocking immune checkpoints enables the immune system to recognize and attack metastatic tumor cells. However, the application of ICB alone cannot trigger the abscopal effect to eliminate metastases. The body needs to activate the immune system to induce tumor cells to produce ICD. Nanoparticles induce ICD to expose the tumor to more TAAs when nanoparticles enter the periphery of the tumor. With the application of ICB, more T cells can infiltrate the tumor. Huang explored a celastrol (CEL) nanoemulsion (Qiu et al., 2021). CEL is recognized for its anti-inflammatory effects *via* suppressing macrophage M1 polarization. In addition, nanoparticles caused an inflammatory response, promoted the recruitment of NK cells in metastatic tumors, and activated tumor-specific T cells to control metastatic tumors. In an experiment involving intravenous injection of CEL nanoemulsion and intraperitoneal injection of anti-PD-L1 in tumor-bearing mice, the CEL nanoemulsion/anti-PD-L1 treatment exhibited a good killing effect, inducing nearly 90% of the total cell apoptosis in treated tumors, with nearly 15-fold or 10-fold more apoptotic cells compared to the control, and anti-PD-L1-treated tumors. After the application of anti-PD-L1, the growth of the primary tumor and the distant tumor was significantly inhibited. Instead of delivering anti-PD1 directly, experimental mice are usually injected intraperitoneally with anti-PD1 to block ICB. Nanoparticles can synergize with ICB to induce stronger abscopal effects after intravenous injection. For examples, upconversion nanoparticles (UCNPs), ROS-sensitive lipid-polymer hybrid nanoparticles, PB nanoparticles and nanoliposomes loaded with pheophorbide A (PhA) have been shown to produce long-term immune memory effects and further enhance the ICB therapeutic outcome (Xu et al., 2017; Hu et al., 2019; Zhou et al., 2020c; Hwang et al., 2020). Tandem peptide nanocomplex (TPNC) and copper chalcogenide nanoparticles carrying CpG can also enhance the therapeutic effect of ICB (Buss and Bhatia, 2020; Cao et al., 2020).

### 3.2 Nanomaterials for blocking the indoleamine 2, 3-dioxygenase pathway

Indoleamine 2, 3-dioxygenase (IDO) is one of the body’s main oxygenases for tryptophan metabolism. It can reduce cellular energy levels, prevent immune cell maturation, and induce T cell apoptosis (Heidari et al., 2020). IDO is the rate-limiting enzyme that metabolizes tryptophan to kynurenine in the body, which regulates the level of metabolism in the body and leads to immune tolerance in the body. IDO is expressed in the cytoplasm of a variety of cells in the TME, DLNs, and peripheral blood. IDO mainly affects the anti-tumor effect of T cells in the following ways. 1) The expression of IDO in tumors is related to the expression of forkhead box P3 (FoxP3) positive T-regs and monocytes in tumor infiltration, and these cells will negatively regulate CD8^+^ T cells in primary tumors and metastases (Meireson et al., 2020). 2) IDO down-regulates the expression of T cells receptors and inhibits T cells proliferation. 3) IDO also affects the function of T cells by reducing the expression of CD107a and granzyme B. 4) IDO also promotes the production of tumor blood vessels (Prendergast et al., 2018). 5) IDO is closely related to the expression of PD-1 and PD-L1 (Dill et al., 2018; Ye et al., 2018). Recent studies have confirmed that the combination of CTLA-4 and CD80/CD86 on APC can induce IDO expression (Walker and Sansom, 2015). IFN-γ produced by T cells and NK cells induces TRC to enter a dormant state through the IDO-Kyn-AhR-p27 cascade, thereby allowing tumor cells to escape immunity (Liu et al., 2017). Therefore, IDO inhibitors have become a hotspot in tumor immunotherapy.

**FIGURE 2:**
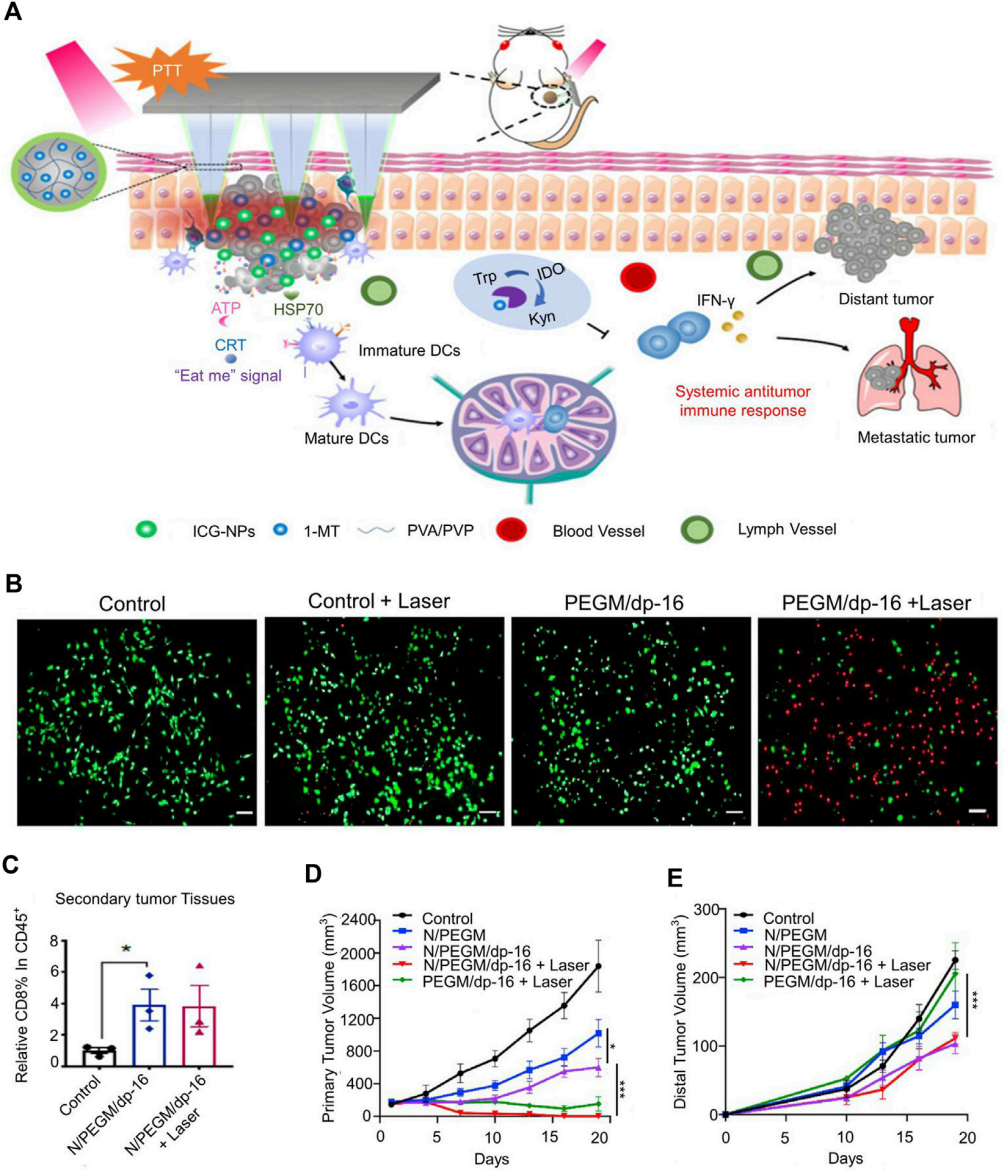
Tumor size-dependent abscopal effect of polydopamine-coated all-in-one nanoparticles for immunochemo-PTT. **(A)** Schematic illustration of the mechanism of anti-tumor immunity. **(B)** The fluorescence images of PANC02 cells co-stained with fluorescein diacetate (FDA, green) and propidium iodide (PI,red) after various treatments. **(C)** Percentages of CD8 + T within the gated CD45^+^ population in primary tumors. **(D)** The tumor volume of primary tumor. **(E)** The tumor volume of distant tumor. Reproduced with permission from (Sun et al., 2020a; Chen et al., 2020).

Most IDO inhibitors cannot function effectively due to their short half-life and poor tumor accumulation (Kuang et al., 2018). Chen et al. (2020) encapsulated indocyanine green (ICG), a photosensitizer, into chitosan nanoparticles (ICG-NPs) (Figure 2A). The zeta potential of ICG-NPs was 47.2 ± 0.3 mV, and the particle size was approximately140 nm. ICG-NPs delivered photosensitizers into tumor cells and effectively regress 80% of tumors in melanoma mice. Sun et al. (2020a) prepared gemcitabine and polydopamine nanoparticles (PGEM/dp-16 NPs) by thin-film aqueous method for loading NLG919, a potent IDO inhibitor. *In vitro* experiments demonstrated that PGEM/dp-16 NP-mediated photothermal therapy (PTT) could efficiently kill cancer cells within 5 min (Figure 2B). IDO inhibitors increased more CD8^+^ T cells after releasing outside tumor cells and reduced T-reg infiltration in the TME. In addition, it can significantly reduce the tumor volume of metastatic tumor models (83%) and reduce the risk of lung metastasis (Figures 2D,E). However, in the early metastatic stage, PGEM/dp-16 NPs did not exhibit a good abscopal effect to regress distant tumors (Sun et al., 2020a). To enhance the delivery of IDO inhibitors, Li et al. (2020b) developed a mesoporous silica nanoparticle (MSN) for the delivery of doxorubicin (DOX) and IDO inhibitors. MSNs were about 70 nm in diameter and released IDO inhibitors outside tumor cells and transport DOX into tumor cells. After systemic administration to tumor-bearing mice, MSN was found to induce tumor regression and reduce the number of lung metastases. To increase the targeting of nanoparticles, Liu et al. (2019) co-encapsulated porphyrin-phospholipid conjugates and IDO inhibitors to form redox-activated liposomes. Redox-activatable liposomes prolonged the existence time of IDO inhibitors in the bloodstream, better target tumor cells. The advantage of redox-activatable liposomes was their ability to released IDO inhibitors in the reducing TME, shown the highest efficacy in inhibiting primary and distant tumors and prevented breast cancer metastasis in an orthotopic 4T1 tumor-bearing mouse model (Liu et al., 2019).

### 3.3 Nanomaterials for delivering cytokines

Cytokines are small-molecule proteins secreted by cells that have the functions of mediating and regulating immune processes. According to their structure and function, they are divided into interleukins, interferons, TNF, colony-stimulating factors, chemokines, growth factors, and so on. This article mainly introduces several cytokines related to the abscopal effect of killing metastatic cancer cells.

IL-12 is a potent pro-inflammatory factor produced by APCs, which induces and enhances cell-mediated immune responses. The target cells of IL-12 are NK cells and T cells, which promote the proliferation of T cells and NK cells and induce the production of γ-IFN. In addition, IL-12 increases the expression of human lymphocyte antigen (HLA) by tumor cells and promote the combination of T cells and antigens to exert anti-tumor effects (Yue et al., 1999). Although IL-12 plays an important role in anti-tumor activity, very small doses of IL-12 cause systemic toxicity (Robertson et al., 1999). The current obstacle to IL-12 treatment is the inability to effectively deliver IL-12 to tumors without causing an increase in systemic IL-12 (Nguyen et al., 2020b). Yao et al. explored a novel photothermal agent and IL-12 gene fragment co-delivery nanoparticle (CSP) (Lin et al., 2021). The hydrodynamic size of nanoparticles increased from 118 to 157 nm. Studies have found that CSP@IL-12 nanocomplex synthesis increased the level of IL-12 expression in B16F10 cells. When CSP@IL-12 nanocomplex was combined with PDT, CSP@IL-12 nanocomplex increased the expression of IL-12 gene fragments in tumors, thereby significantly increased the amount of IL-12 in the TME. CSP@IL-12 nanocomplex achieved good tumor regression, and produced the abscopal effect to kill metastatic cancer cells. In order to increase the targeting of nanoparticles to TME, Sun et al. (2020b) designed a novel nanosystem based on TAM charge-reversal polymetformin (PMet), which simultaneously delivered DOX and IL-12 gene fragments. The mechanism of increased TAM targeting lies in the synergistic encapsulation of thiolated hyaluronic acid (HA-SH) into PMet nanosystems to facilitate precise TME targeting. PMet nanoparticles with an average particle size of 192.3 nm showed improved anti-tumor and antimetastatic activity in a mouse model of breast cancer lung metastasis. However, PMet nanoparticles formed aggregates with serum components shortly after incubation. Such aggregates do not seem to be conducive to the long-term antitumor effect of PMet nanoparticles *in vivo*. In order to further improved the carrying efficiency, Antonio et al. used Layer-by-layer (LBL) assembly to load IL-12 gene fragments onto the surface of glyceroliposomes with poly-L-arginine (PLR) as a polymer coating for reducing systemic toxicity (Barberio et al., 2020). For the external layer, both hyaluronic acid (HA) and poly-l-glutamic acid (PLE) were chosen to produce a dense negative charge. LBL-NPs were 80–120 nm in diameter, and each LbL-NP contained approximately 50 IL-12 molecules. Reduction of IL-12 systemic toxicity and induction of abscopal effects were observed in a ovarian cancer tumor-bearing mice.

IL-2 is another cytokine of the interleukin family, also known as T cells growth factor. IL-2 binds to the IL-2 receptor and affects the proliferation, activation, and differentiation of immune cells (Raker et al., 2020). IL-2 has a certain effect on the proliferation of effector T cells, but it is not necessary for the proliferation of effector T cells. Different doses of IL-2 activate different types of immune cells (Raker et al., 2020). In the presence of low-dose IL-2, IL-2 receptors are mostly activated on Tregs; under medium- and high-dose IL-2 conditions, also lower-affinity IL-2 receptors are bound on effector T cells and NK cells (Raker et al., 2020). Therefore, it seems advantageous to maintain high-dose IL-2 levels in the tumor microenvironment. However, excessively high doses of IL-2 regulate tumor-reactive CD8^+^ T cell exhaustion by activating the aryl hydrocarbon receptor (Liu et al., 2021). The short lifespan of IL-2 has also become an obstacle to its use as an immunotherapy drug. To increase the half-life of IL-2 in the body, Votavova et al. (2015) explored combining IL-2 gene fragments with a co-polymer to make it a nanoparticle with an IL-2 agonist effect. Each IL-2 molecule carries 2 methacrylamides (HPMA). This co-polymer can prolong the half-life of IL-2 in the body and effectively control the concentration of IL-2, thereby avoiding the toxicity caused by excessive IL-2 concentration (Votavova et al., 2015). However, the biological activity of IL-2 modified by this co-polymer is weaker than that of normal IL-2. Although it causes the increase of CD8^+^ T cells and NK cells in the body, the anti-tumor effect needs to be further improved. Yin et al. co-delivered DOX and IL-2 with chitosan nanoparticles (Wu et al., 2017). Chitosan nanoparticles carrying DOX and human recombinant IL-2 solved the toxicity problem of DOX and IL-2 in the bloodstream. However, it has been found that local delivery of IL-2 increases the expression of T-regs, which was detrimental to the antitumor effect (McHugh et al., 2015; Horwitz et al., 2019). To reduce the local effect of T-regs on the antitumor effect without reducing the effect of IL-2, Jason et al. developed nanoscale liposomal polymeric gels (nLGs) for the delivery of IL-2 and TGF-β receptor-I inhibitors (Park et al., 2012). Elimination of TGF-β reverses TAM immunosuppression and enhanced antitumor immune responses. After 3 days of systemic administration, nLGs were able to maintain 38% ± 8% of initial concentrations in a mouse model of metastases. This indicates that nLGs not only increase the concentration and half-life of IL-2, but also effectively reversed the immunosuppressive environment. In another study, Sharma et al. (2020) used PEG and IL-2 to prepare a prodrug of IL-2 that could directly eliminate T-regs in TME. It selectively induced the release of cytokines on tumor-infiltrating T cells and selectively depleted Tregs in the tumor without causing changes in the number of Tregs outside the tumor (Sharma et al., 2020). Although this treatment has a good limiting effect on tumors, it has not been reported to have an abscopal effect on metastases.

Although there are not many studies on cytokine-regulated immunotherapy to trigger the abscopal effect, the effectiveness of cytokines is obvious. Nanomaterials can enhance cytokine targeting and dose sensitivity. We believe that nanomaterials to regulate cytokines to trigger the abscopal effect will have very good application prospects.

### 3.4 Nanomaterials for depleting tregs

Tregs are cells that play an inhibitory role in the body. For example, Tregs produce immune tolerance by producing large amounts of TGFβ, IL10, IL35, and other factors (Schmidt et al., 2012). Tregs are produced by continuous antigen stimulation of T cells and are induced by TGF-β, and they exert their effects through TGF-β and other cytokines (Turner et al., 2020). After Tregs are activated, cytokine immunosuppression is non-antigen specific. This non-antigen specificity is not restricted by MHC and can inhibit the proliferation and activation of CD8^+^ T cells. This is the main reason for limiting distant tumor suppression (Legoux et al., 2015). The γ-IFN produced when T cells exert anti-tumor activity can regulate the development and differentiation of Tregs through interferon regulatory factor 4 (IRF4) (Alvisi et al., 2020). In the context of γ-IFN and TGF-β, peripheral CD4^+^CD25-T cells can be transformed into CD4^+^CD25 + Treg, accompanied by the up-regulation of Foxp3. CD25 is the receptor of IL-2. Tregs in tumors up-regulate the expression of the IL-2 receptor, inhibit other immune cells from binding to IL-2, and affect the activation of immune cells (DeOca et al., 2020).

An increase in the number of effector T cells will also cause an increase in the number of Tregs. Besides T cells, the function of APCs is also regulated by Tregs. The combination of Tregs and APC can cause surface molecules to peel off and inhibit the antigen presentation of APC cells (Raffin et al., 2020). DCs produce CCL22, bind to the CCR4 receptor, and regulate the migration of Tregs to the TME (Röhrle et al., 2020). Therefore, how to reduce the number of Tregs around the metastatic tumor has become a key problem in the field of tumor immunotherapy.

In addition to the aforementioned cytokine delivery that eliminates T-regs, direct depletion of T-regs in TME also enhance antitumor responses. Kohno et al. (2020) injected diphtheria toxin into tumor cells to deplete Tregs in the spleen, blood, and lymph nodes of DEREG transgenic mice after irradiation, and the survival time of the mice was prolonged. Diphtheria toxin depleted Foxp3+ Tregs in these transgenic mice. This reduces T-reg in the distal tumor microenvironment and enhances the distant effect triggered by the immune response. Depletion of Tregs induced the activation of systemic T cells and produce cytokines and cytolysins. Although depletion of Tregs throughout the body caused tumor cell regression, it also caused severe inflammation in the body (Lui et al., 2020). In one study, a homemade curcumin analog (CA) was encapsulated in α-lactalbumin (α-LA), and the Treg cell-specific antibody (mAb), as a therapeutic agent, was linked to the drug-loaded protein *via* matrix metalloproteinase-responded peptide (P) to form CA@α-LA-P-mAb NPs (Du et al., 2020). The diameter of CA@α-LA-P-mAb NPs were 175.0 ± 8.4 nm. It was shown that CA@α-LA-P-mAb NPs could mediate mitochondrial apoptosis, but CA@α-LA-P-mAb NPs had no toxicity to normal cells. Under the CA@α-LA-P-mAb, tumors were dramatically reduced, and the tumor inhibition rate was 75.0%. In conclusion, targeting the depletion of Tregs delayed tumor growth. In addition, increasing the ROS of TME reduced the production of T-regs. Catalase loaded onto polydopamine -ICG nanoparticles and pH-sensitive polymethacrylate-coated CaO_2_ nanoparticles enhanced ROS generation in tumors, resulting in the reduction of intratumoral FOXP3+ regulatory T cells (Table 1) (Nicholas et al., 2021; Sun et al., 2021).

## 4 Nanomaterials for regulating myeloid-derived suppressor cells to trigger abscopal effect

Myeloid-derived suppressor cells (MDSCs) in the body mainly exist in the bloodstream and around tumors. They can be divided into MDSCs from mononuclear sources (i.e., M-MDSCs) and those from granulocyte sources (i.e., G-MDSCs). G-MDSCs mainly exist in peripheral lymphoid organs, and M-MDSCs mainly exist around tumors. M-MDSCs promote tumor cells invasion, angiogenesis, and metastasis formation and inhibit anti-tumor immunity (De Cicco et al., 2020). MDSCs in the bone marrow are recruited to peripheral lymphoid organs and tumor sites through growth factors secreted by cancer cells (Law et al., 2020). MDSCs progenitor cells are usually necessary to restore tissue homeostasis after infection and traumatic stress. However, MDSCs cannot differentiate into immunogenic DCs or inflammatory macrophages in the TME. Once recruited, MDSCs promote the formation of abnormal tumor blood vessels, destroy the antigen presentation of DCs, inhibit the functions of T cells and NK cells, and polarize TAMs into the M2 phenotype (Goldszmid et al., 2014; Kumar et al., 2016a). In addition, MDSCs reduce the secretion of γ-IFN by secreting IL10 and TGF to recruit Tregs (Sacchi et al., 2018). In recent years, inhibiting MDSCs has emerged as a potentially promising cancer treatment. Approaches include 1) depleting MDSCs, 2) inhibiting the recruitment of MDSCs to the tumor site, 3) inhibiting the inhibitory activity of MDSCs, and 4) promoting the differentiation of MDSCs (De Cicco et al., 2020).

### 4.1 Nanomaterials for polarizing myeloid-derived suppressor cells

MDSCs are immunosuppressive cells, mainly including immature monocytes and granulocytes. Immature DCs are also a type of MDSCs. Studies have shown that ROS inhibited MDSCs differentiation (Kusmartsev and Gabrilovich, 2003). All-trans retinoic acid down-regulated the effect of ROS and regulated MDSCs differentiation by up-regulating glutathione (GSH) synthesis and down-regulating ROS content in cells (Nefedova et al., 2007). Studies have found that all-trans retinoic acid reduces MDSCs and improves the effect of immunotherapy treatment for sarcoma (Kusmartsev et al., 2003; Long et al., 2016).

The hypoxic tumor environment leads to the down-regulation of STAT3, which controls the differentiation of MDSCs to TAMs (Kumar et al., 2016b). Elimination of STAT3 expression by conditional knockout mice or selective STAT3 inhibitors can significantly reduce MDSCs expansion and T cells responses (Kortylewski et al., 2005). As an inhibitor of STAT3, docetaxel reduces tumor-induced MDSCs, TAMs, and endothelial cells infiltration into tumors, thereby inhibiting angiogenesis, tumor growth, and metastasis (Tsai et al., 2017). Interestingly, it has been clinically reported that docetaxel administered as a chemotherapeutic drug can trigger the abscopal effect to promote the regression of urothelial cancer (Ishiyama et al., 2019). PEGylated liposomes loaded with DOX nanoparticles carrying docetaxel increased the intratumoral concentration of the drug and reduce the concentration of the drug in the bloodstream, thus reducing the toxic side effects of the drug (Atrafi et al., 2020). PEGylated liposome loaded with doxorubicin was the first FDA-approved nanomedicine. It has been shown in phase one clinical trial that it increased plasma concentrations. In addition, there have been related reports on the role of TLRs and granulocyte-macrophage colony-stimulating factor (GM-CSF) in inducing MDSCs differentiation (Shirota et al., 2012; Kong et al., 2020; Long et al., 2020; Zamorina et al., 2020). These reports produced antitumor effects in primary tumor models. However, there are not many studies on the targeting of nanomaterials for MDSCs-induced immunotherapy to trigger the abscopal effect.

### 4.2 Nanomaterials for blocking MDSC infiltration

Many pathways affect MDSCs apoptosis. IL4Rα and STAT6 play important roles in the activation of MDSCs and the maintenance of theirs inhibitory activity by regulating the expression of ARG1 and the secretion of TGF-β (Roth et al., 2012). IL4 regulates ARG-1 transcription through the IL4Ra-STAT6 pathway and increases L-arginine depletion, reducing T cells’ survival rate (Grzywa et al., 2020). RNA aptamer specifically recognizes IL4Ra allowing it to target MDSCs, and induces MDSCs apoptosis by blocking signals from IL4Ra (Roth et al., 2012). However, IL4Ra is also expressed on B cells, T cells, and TAMs. Therefore, RNA apoptosis is not very specific to MDSCs apoptosis.

CXCR2 is the G protein-coupled receptor of human (C–X–C motif) chemokine. The expression of CXCR2 in cancer cells drives proliferation, invasion, and migration (Steele et al., 2016). However, CXCR2 is expressed on MDSCs but less expressed on tumor cells (Steele et al., 2016). Knocking out CXCR2 or CXCR2 inhibitors generate more T cells and reduce pancreatic cancer metastasis (Steele et al., 2016; Sun et al., 2019). Therefore, exploring nanoparticles to inhibit MDSCs by targeting CXCR2 inhibitors in tumors is a promising cancer treatment.

Tumor hypoxia has also been shown to recruit MDSCs. Although chemotherapeutic drugs can directly clear MDSCs, clearing MDSCs in normal tissues has the risk of inducing immunodeficiency (Zuo et al., 2020). Therefore, targeted elimination of MDSCs in the TME is a promising therapeutic modality. In a study, silica nanoparticles carrying mitochondrial respiration inhibitors improved hypoxia around tumors. The silica nanoparticles blocked the infiltration of MDSCs into the tumor and enhanced the anti-tumor effect (Tuettenberg et al., 2016; Zuo et al., 2020). He et al. developed nanoparticles (PLT NPs) using phenylboronic acid-modified low molecular weight heparin and tocopheryl succinate (Lu et al., 2021). The average particle size of PLT-NPs were 140 nm. PLT NPs could significantly improve the immune microenvironment of pancreatic tumors and inhibited spontaneous metastases. PLT NPs will not damage red blood cells as the dose increases. When such nanoparticles were injected into the body, it was observed that nanoparticles accumulated at the tumor site and reduced the infiltration of MDSCs around the tumor (by 5.02%) on the 18th day. Additionally, phenylboronic acid-modified nanoparticles reduced the number of metastases, and non-toxic heavy metal-oxo cluster secondary building units (SBUs) and photosensitizing bridging ligands enhanced the immunotherapy of metastatic cancers (Ni et al., 2019).

Although MDSCs have been demonstrated as suppressor cells in TME. However, current studies on regulating the abscopal effects of MDSCs are uncommon. This may be related to the potential of MDSCs to differentiate into other cells. The mechanism of the abscopal effect remains elusive. Therefore, it seems difficult to regulate MDSCs alone to trigger abscopal effects.

## 5 Nanomaterials for regulating targeting tumor-associated macrophages to trigger abscopal effect

Macrophages play an indispensable role in cancer immunotherapy. Macrophages are mainly divided into M1 type and M2 type (Mantovani and Locati, 2013). M1-type macrophages activate CD8^+^ T cells and NK cells by presenting TAAs and producing cytokines, which can kill metastatic cancer. M2-TAMs secrete a variety of inhibitory cytokines, such as IL6, IL10, and TGFβ, and chemokines, such as CCL4, CCL5, and CCL22, to induce Tregs to migrate to the TME (Qian et al., 2011). However, macrophages are more likely to differentiate into the M2 phenotypes that promote tumor growth due to their own plasticity (Mantovani et al., 2017). Like MDSCs, M2-TAMs suppress the immune system around the tumor, induce tumor angiogenesis, and promote tumor progression.

### 5.1 Nanomaterials for inducing the polarization of macrophages from M2 to M1

TLR7 and TLR9 are expressed on the surface of macrophages but not on the surface of tumor cells (Trinchieri and Sher, 2007). TLR agonists induce TAMs to differentiate into the M1 phenotype and trigger the abscopal effect (Shan et al., 2020; Han et al., 2021). However, due to the poor pharmacokinetics of TLR agonists, even intratumoral administration cannot achieve the best results. Zhang et al. (2020) designed a thermosensitive liposome with TLR agonists, loaded them into phosphocholine liposomes. Compared with simple intratumoral injection, thermosensitive liposomes induced more M1-type TAMs, whereas there was almost no change in M2-TAMs. The application of thermosensitive liposomes obtained longer survival rates and better long-term immune memory (Zhang et al., 2020). However, the loading rate of the thermosensitive liposomes was not enough to trigger a powerful abscopal effect. Rodell et al. explored β-cyclodextrin nanoparticles (CDNPs) for carrying TLR agonists. CDNPs were able to aggregate within tumors and were specifically captured by macrophages 24 h after injection and could more strongly differentiate M2-TAMs from M1-TAMs (Rodell et al., 2018). Unlike the high macrophage affinity of β-cyclodextrin, apoptotic bodies (ABs) as a delivery vehicle has demonstrated a strong tumor accumulation potential. Encapsulation of ABs into nanoparticles is beneficial to increase nanoparticle deposition in tumor cells. Zheng et al. (2020a) conjugated CpG to gold-silver nanorods (AuNR) and loaded them into Abs (AuNR-CpG/AB). The loading efficiency of AuNR-CpG/ABs were 3.76% ± 0.55%. More than 80% of AuNR-CpG/ABs was deposited in miscellaneous tumor cells after intravenous injection in tumor-bearing mice.

Yook et al. explored Cetuximab-targeted gold nanorods (CTX-AuNR) (Emami et al., 2021). CTX-AuNR was about 11.2 ± 1.8 nm wide and 48.2 ± 2.5 nm long. The viability of BT-20 spheroids after photoimmunotherapy (PIT) of NT-AuNR near-infrared irradiation (NIR) was much higher than that of BT-20 spheroids grown with TAM (98.6% ± 0.6%). The addition of Bi(NO_3_)_3_ into nanoparticles acted as a photosensitizer (PT) and enhanced the abscopal effect of PTT-triggering immune effects (Emami et al., 2021). Bi-based nanomaterials loaded with doxorubicin and hyaluronic acid induced the conversion of TAMs from M2 to M1 (Emami et al., 2021). Hyaluronic acid nanoparticles loaded with MnO_2_ not only improved the local hypoxic state of the tumor but also induced the conversion of TAMs from M2 to M1 (Song et al., 2016; Parayath et al., 2018). In addition, macrophage colony-stimulating factors (M-CSFs) were key regulators of monocyte differentiation and formation and tissue-resident macrophage activity. Mao et al. used calcium carbonate as a cross-linking agent to load M-CSF into polyethylene glycol-polyglutamic acid nanoparticles (Mao et al., 2019). Nanoparticles have high drug loading efficiency (41.9% ± 12.4%). *In vitro* experiments showed that TAM can effectively accumulate within 1 h of injection. Nanoparticles reverse TAM phenotype and increase antitumor effect in tumor-bearing mice.

### 5.2 Nanomaterials for repolarizing macrophages into tumors

As a chemokine, chemokine (C-C motif) ligand 2 (CCL2)induces the migration of inflammatory cells and immune cells in the body. CCR2, as the receptor of CCL2, is mainly expressed in monocytes and NK cells (Hao et al., 2020). In addition, monocyte chemoattractant protein-1 (MCP-1) can also function through the CCR2 signaling pathway. CCR2 and its ligand Mcp1 pathway stimulate tumor cell proliferation (Ding et al., 2019). MCP1-CCR2 drives monocytes into the TME, which inhibits the anti-tumor activity of T cells. However, because Mcp1 inhibitors cannot be maintained in the body for a long time, the injection of Mcp1 inhibitors alone cannot cause sufficiently strong anti-tumor activity. Trac et al. (2020) explored the use of micelles to target CCR2 on monocytes and introduced apoptotic peptides into the micelles. These micelles were able to bind to monocytes in the body and induce their apoptosis, reduce the infiltration of TAMs into tumors, and promote the infiltration of T cells into the tumor, thereby enhancing the effect of immunotherapy. Shen et al. (2018) wrapped siCCR2 fragment (a siRNA for blocking CCR2 expression) in poly (ethylene glycol)-block-polylactide nanoparticles (PLG-PLA), which caused siCCR2 to target monocytes. SiCCR2 fragment-encapsulated polyethylene glycol-lactide nanoparticles. The diameter of cationic nanoparticles were 126.8 ± 15.6 nm. Cationic nanoparticles had better targeting of monocytes than neutral nanoparticles in breast cancer-bearing mice, and some of them blocked the expression of CCR2 on monocytes, blocked the infiltration of monocytes into tumor cells and reversed the immunosuppressive state in the TME. Moreover, cationic polymeric nanoparticles can promote tumors to produce CCL2 to recruit more immune cells, thereby enhancing the efficacy of chemotherapeutics (Leuschner et al., 2011; Liang et al., 2018b; Shen et al., 2018). However, these nanoparticles do not show that the abscopal effect can be enhanced.

**FIGURE 3:**
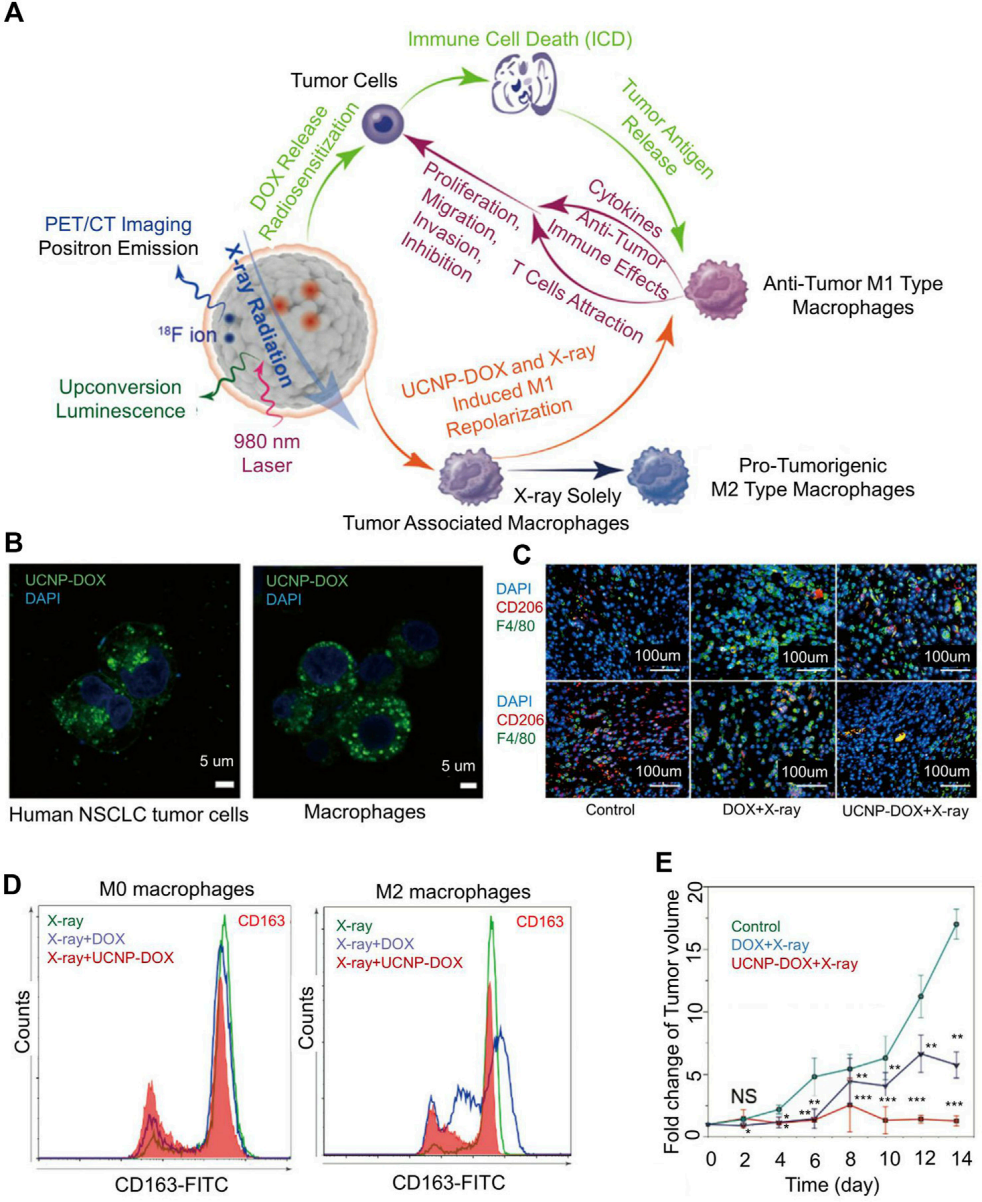
Mesoporous Bi-containing radiosensitizer to repolarize TAMs and boost abscopal effect. **(A)** The schematic of mesoporous Bi-containing radiosensitizer. **(B)** Confocal images of internalization of nanoparticles by lung cancer cells and macrophages. **(C)** After enhanced chemoradiotherapy of upconversion nanophosphor (UCNP) loaded with doxorubicin (UCNP-DOX) and X-ray, the tumor tissues showed intense CD68 and weak CD206 signals, showing the increased M1 macrophage level and down-regulated M2 macrophage level. **(D)** Polarization of macrophages in different treatment groups. **(E)** Fold change of tumor volume in all groups. Reproduced with permission from (Qin et al., 2020).

Because of the unique biocompatibility of bismuth (Bi) and the ability of bismuth as PS to enhance PDT, Bi-based nanomaterials have been reported for bioapplications (Qin et al., 2020). Bi-based mesoporous upconversion nano-supported DOX. Lu explored a Bi-based mesoporous upconversion nanophosphor (UCNP) loaded with doxorubicin (UCNP-DOX) (Figure 3) (Qin et al., 2020). Bi(NO_3_)_3_ was dissolved in 20 ml of ethylene glycol and stirred until a clear solution formed. After adding DOX and stirring for 48 h, the solvent was evaporated. The diameter of UCNP-DOX was 85 nm. *In vitro* experiments show nanoparticles can be internalized by tumor cells and macrophages (Figure 3B). In the tumor-bearing mouse models of lung and colon cancer, after UCNP-DOX and X-ray treatment, the signal of CD68 (M1-TAM surface marker) in tumor tissue was strong, and the signal of CD206 (M2-TAM surface marker) was weak. The phenomenon showed that the level of M1 macrophages was increased, and the level of M2 macrophages was down-regulated (Figures 3C,D). The combination of nanoparticles and radiotherapy caused more M1-TAM than M2-TAM and delayed tumor growth (Figure 3E).

## 6 Nanomaterials for regulating cancer-associated fibroblast to trigger abscopal effect

In recent years, the role of cancer-associated fibroblast (CAF) in tumors has been recognized, and CAF has become a new target for cancer treatment. CAF inhibits tumor cell proliferation through TGF-β and IL-6 (Louault et al., 2020). However, CAF recruits inflammatory cells to reshape the extracellular matrix and promotes tumor blood vessel growth by secreting angiogenic factors (Erez et al., 2010). Furthermore, CAF promotes cancer cell invasion and metastasis through TGF-β and IL-32 and recruits MDSCs through CCL2, IL-1-β, monocytes, and so on to inhibit the anti-tumor effect of CD8^+^ T cells (Louault et al., 2020). Studies have confirmed that platelet-derived growth factor (PDGF) and TGFβ were the main factors for CAF activation (Micke and Ostman, 2005). Dasatinib treatment partially reversed the CAF phenotype in lung cancer tissues and reduce its ability to promote tumor proliferation *in vitro* (Haubeiss et al., 2010). Zhang et al. (2021) developed gold nanoparticles with a diameter of 20 nm that inhibited the expression of fibrin on the surface of CAF and thus inhibited the activation of CAF (Hossen et al., 2019). After gold nanoparticles were internalized by CAF, RT will caused more CAF damage (Bromma et al., 2020). In addition, PTT reshaped the tumor extracellular matrix and reduced the amount of CAF in the TME (Tan et al., 2019; Zheng et al., 2020b). The oxidized nanoparticles developed by Ferraz had the characteristics of fast and efficient cells internalization and low cytotoxicity (Ferraz et al., 2020). Ferric chloride and ferric sulfate were coprecipitated in sodium hydroxide solution and coated with trisodium citrate solution at 90°C for 30 min. Nanoparticles had an average diameter of 8.9 ± 2.4 nm in diameter. The oxidized nanoparticles were able to induce hyperthermia and human fibroblast cell death through apoptosis *in vitro*. They can induce heat generation and promote CAF apoptosis in a variable magnetic field. Although The oxidized nanoparticles promoted CAF apoptosis *in vitro*, it has not been verified *in vivo*.

**FIGURE 4:**
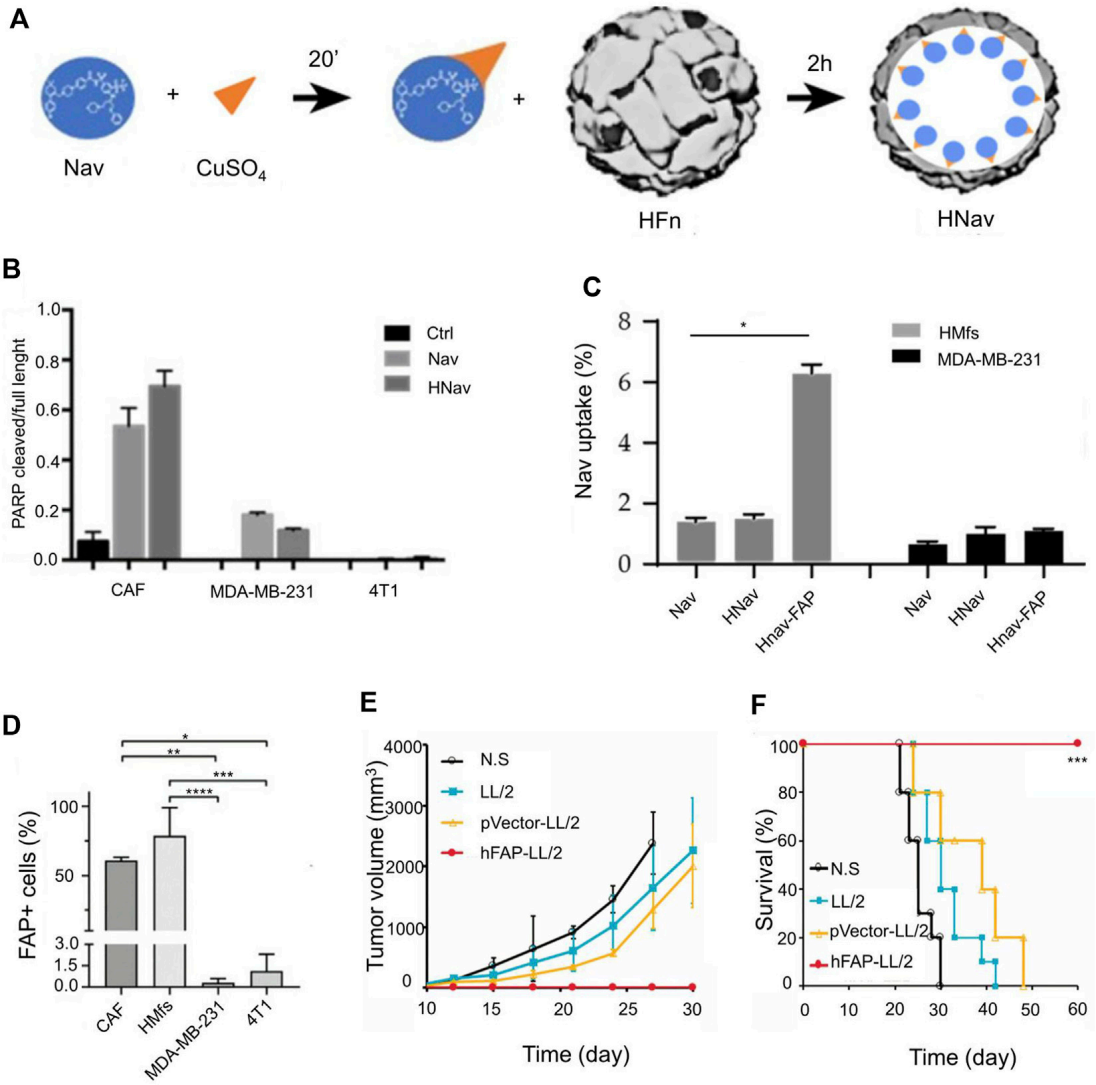
Engineered H-ferritin nanocage targets tumor-associated fibroblasts to induce anti-tumor immune response. **(A)** Development of H-Ferritin nanocages loaded with Navitoclax (Hnav) and HNav-fibroblast activation protein (FAP). **(B)** Calculated lysis of PARP-1 when CAF cells and breast cancer cells are incubated with 1 µM Nav or HNav. **(C)** Human activated myofibroblasts (HMFs) and breast cancer cellular uptake of Nav. **(D)** FAP expression in CAFs, HMfs, MDA-MB-231, and 4T1 cells. **(E)** The tumor growth curves in different treatment groups. **(F)** The survival rate in different treatment groups. Reproduced with permission from (Chen et al., 2019; Sitia et al., 2021).

Fibroblast activation protein (FAP) is important for CAF activation. Currently, FAP is the main target for CAF regulation (Lindner et al., 2019). Corsi et al. developed H-ferritin nanocages by utilizing metal ion affinity method; these were loaded with navitoclax and functionalized with FAP antibody fragments for targeting CAF (Figure 4A) (Sitia et al., 2021). HFn may contribute to increased Nav intratumoral accumulation due to HFn’s natural tumor homing and nanoparticle-mediated enhanced permeability and EPR effects. The poly ADP-ribose polymerase (PARP) cracking rate increased after treatment with H-ferritin nanocages (Figure 4B) (Chen et al., 2019). After encapsulation of FAP, H-ferritin nanocages exhibited high endocytosis efficiency (Figure 4C). FAP is highly expressed in CAF (Figure 4D). After the targeted elimination of CAF, it was observed that the growth of both the primary tumor and the distal tumor was delayed (Figures 4E,F). In addition, delivering oxygen to the TME is also a way to reverse the inhibitory TME. Duan et al. (2021) encapsulated perfluorocarbons (PFCs) with PLGA nanoparticles to improve peritumoral hypoxia levels. The PLGA nanoparticles were 110 nm in diameter. The addition of PFC can maintain the stability of nanoparticles. In addition, the addition of lignan-derived compounds to the nanoparticles was beneficial to promote the production of IL-25 by CAFs. IL-25 promoted tumor cell apoptosis. Regression of the primary tumor and metastases was observed 17 days after the nanoparticles were injected into the mice. However, whether PFC@PLGA reduces the number of CAFs has not been verified.

## 7 Nanomaterials for regulating NK cells to trigger abscopal effect

NK cells are natural immune cells in the body, and they are the first line of defense against infection and tumors. Unlike T cells, NK cells do not need antigen stimulation to produce anti-tumor effects, nor do they need to secrete cytokines to regulate immune responses. NK cells secrete perforin and granzymes to induce cell death directly. Once tumor cells are recognized and attacked by NK cells, their efficiency is greatly reduced. However, NK cells alone cannot effectively eliminate tumors.

**FIGURE 5:**
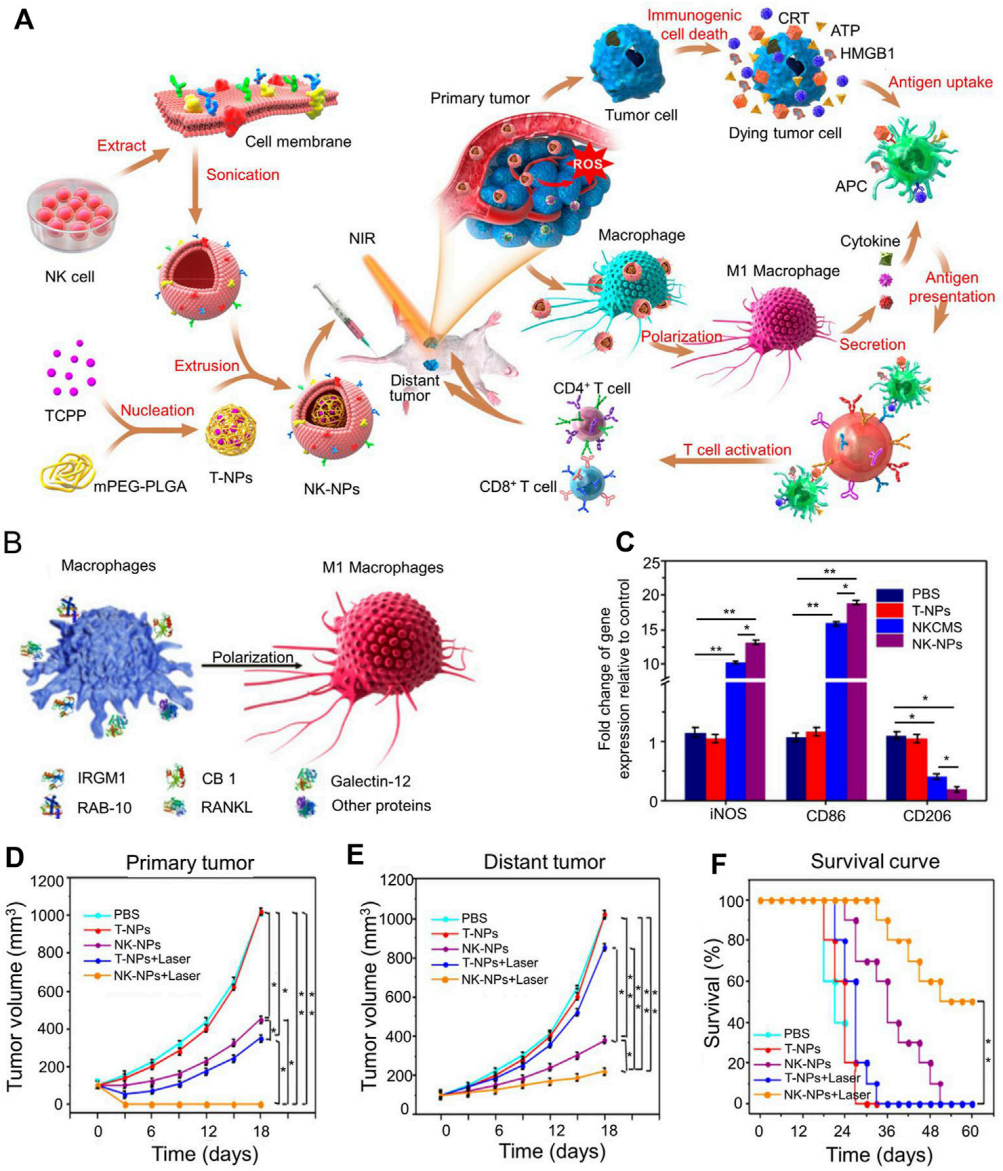
Immunotherapy based on NK cell membrane coated nanoparticles for the effective inhibition of abscopal tumor growth. **(A)** Schematic illustration of NK cell-membrane-cloaked nanoparticles for PDT-enhanced cell-membrane immunotherapy. **(B)** Human NK-NPs and pro-inflammatory M1-macrophage polarization in THP-1 cells. **(C)** The signs of M1-macrophage activation *in vitro*. **(D)** The tumor volume of primary tumor. **(E)** The tumor volume of distant tumor. **(F)** The survival rate of all groups. Reproduced with permission from (Deng et al., 2018).

NK cells can target tumor cells through proteins such as receptor activators of nuclear factor-kappa B ligand (RANKL) or DNAX accessory molecule (DNAM-1) present in the NK cell membrane. Cai designed nanoparticles coated with NK cell membranes, which produced an abscopal effect to inhibit tumor growth (Figure 5) (Deng et al., 2018). Extracted NK cells membranes were extrusion-coated onto polymer nanoparticles loaded with PT. The nanoparticles were polymerized from mPEG-PLGA loaded with benzoic acid and had an average particle size of 80 ± 1.5 nm. In anti-tumor immunotherapy, NK cells induced the polarization of pro-inflammatory M1-macrophages and targeted tumor cells through proteins (such as RANKL or dNaM-1) present on the cell membranes of NK cells (Figure 5B). A schematic illustration of NK cells membrane-cloaked nanoparticles for PDT-enhanced cells membrane immunotherapy was shown in Figure 5A. NK cells membrane-cloaked nanoparticles increased the expression of the M1 macrophage’s markers iNOS and CD86 and decreased the expression of the M2 macrophage marker CD206 (Figure 5C). NK cells membrane-cloaked nanoparticles also increased the production of M1 macrophage-related cytokines (TNF-α, IL-6, and IL-12). NK-NPs were able to eliminate primary tumor growth and produce the abscopal effect to inhibit the growth of distant tumors (Figures 5D,E). NK cells membrane-cloaked nanoparticles also improved the survival time of experimental mice (Figure 5F). Although NK cells have a strong killing function, they need to activate the immune system to kill tumors.

## 8 Conclusion and perspectives

Cancer metastasis is the leading cause of death in cancer patients, as there are no effective clinical treatments for metastatic cancer. In recent years, abscopal effects associated with immunotherapy have been observed. However, immunotherapy has only been effective in triggering the abscopal effect in basic experiments. Moreover, immunotherapy can cause harm to the body by introducing excessive inflammatory factors and cytokines. Therefore, nanomaterial-based delivery methods for immunotherapeutic agents have been developed. With the continued development of nanomaterials, the immune-induced abscopal effect may become more feasible for the treatment of metastatic tumors. Immune cells play a key role in killing metastatic cancer cells. Nanomaterials can be designed to induce tumor antigen exposure, lead to DC and T cell maturation, suppress immunosuppressive cells, increase the number of T cells around metastases, and in general reverse the immunosuppressive microenvironment to provide favorable conditions for the removal of metastases. In preclinical studies, nanomaterials have been proven to enhance the abscopal effect and reduce the negative effects (e.g., immune off-target and irAEs) of immunotherapy. Nanomaterials also offer a promising pathway for the combination of immunotherapy and other treatment methods such as RT and chemotherapy.

However, TAM is a complex system. How to design nanomaterials that target tumor cells is important. At present, acid-responsive, hypoxia-responsive, and metal-responsive nanomaterials have been reported by many researchers. These nanomaterials achieved good results in mice. Although the specific mechanism of the abscopal effect is still unclear, the abscopal effect of nanomaterials triggered by immunotherapy has also been confirmed in animal experiments. However, abscopal effects are not found in all metastases. This phenomenon is still observed in only a minority of tumors. Although we have made breakthroughs in studying primary tumors with different nanomaterials, the study of metastases still needs to be explored further.
